# Fiber Bragg Grating Sensors for Harsh Environments

**DOI:** 10.3390/s120201898

**Published:** 2012-02-10

**Authors:** Stephen J. Mihailov

**Affiliations:** Communications Research Centre Canada, 3701 Carling Avenue, P.O. Box 11490, Station H, Ottawa, ON K2H 8S2, Canada; E-Mail: stephen.mihailov@crc.gc.ca; Tel.: +1-613-998-2721; Fax: +1-613-993-7139

**Keywords:** fiber Bragg grating, sensor, optical sensing, harsh environment sensing

## Abstract

Because of their small size, passive nature, immunity to electromagnetic interference, and capability to directly measure physical parameters such as temperature and strain, fiber Bragg grating sensors have developed beyond a laboratory curiosity and are becoming a mainstream sensing technology. Recently, high temperature stable gratings based on regeneration techniques and femtosecond infrared laser processing have shown promise for use in extreme environments such as high temperature, pressure or ionizing radiation. Such gratings are ideally suited for energy production applications where there is a requirement for advanced energy system instrumentation and controls that are operable in harsh environments. This paper will present a review of some of the more recent developments.

## 1. Introduction

Sensing technologies based on optical fiber have several inherent advantages that make them attractive for a wide range of industrial sensing applications. They are typically small in size, passive, immune to electromagnetic interference, resistant to harsh environments and have a capability to perform distributed sensing. Because of their telecommunication origins, fiber optic-based sensors can be easily integrated into large scale optical networks and communications systems.

**Figure 1.**
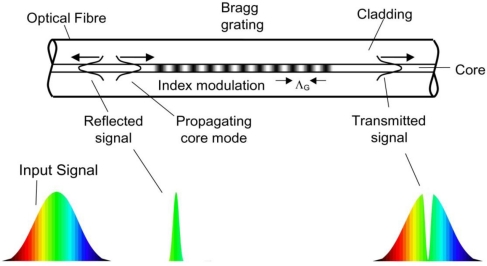
Schematic diagram of an FBG having an index modulation of spacing *Λ_G_* inside a single-mode optical fiber.

Although developed initially for the telecommunications industry in the late 1990’s, fiber Bragg gratings (FBGs) are increasingly being used in sensing applications and are enjoying widespread acceptance and use. The FBG is an optical filtering device that reflects light of a specific wavelength and is present within the core of an optical fiber waveguide. The wavelength of light that is reflected depends on the spacing of a periodic variation or modulation of the refractive index that is present within the fiber core. This grating structure acts as a band-rejection optical filter passing all wavelengths of light that are not in resonance with it and reflecting wavelengths that satisfy the Bragg condition of the core index modulation. The Nobel Laureate Sir William Lawrence Bragg established the Bragg law in 1915, describing with a simple mathematical formula how X-Rays were diffracted from crystals. The Bragg condition, when applied to fiber Bragg gratings, states that the reflected wavelength of light from the grating is *λ_B_* = 2·*n_eff_* ·*Λ_G_* where *n_eff_* is the effective refractive index seen by the light propagating down the fiber, and *Λ_G_* is the period of the index modulation that makes up the grating. A diagram of an FBG is shown in Figure 1. Typically, the modulation of the core refractive index is created by photoimprinting a hologram in the photosensitive glass core of the fiber. Like a photographic film, the core of standard telecommunication optical fiber was found by researchers at the Communications Research Centre Canada to be photosensitive [1]. They found that Germanium, the element that is commonly used to raise the refractive index of silica in the core region of an optical fiber, when exposed to high intensity visible or ultraviolet (UV) light, further increased the core refractive index. By modulating the high intensity light along the length of the fiber core, a modulated change in the refractive index of the fiber core was realized. Typically, this spatial modulation of the writing beam is realized by transmitting the UV light through a special transmission diffraction grating that is precisely etched to null the transmitted zero order [2]. This diffraction grating is often referred to as a phase mask. The light exiting the mask is mostly coupled into the resulting ±1 orders. The interference of these transmitted orders causes a spatial modulation of the beam that is photo-imprinted along the length of the core of the optical fiber.

In addition to telecommunication applications, the FBG is also ideally suited for sensing purposes especially those requiring monitoring of temperature and stress. Optical components and sources already developed for use in the telecom industry can be utilized for sensing network applications. Being a completely optical device, an FBG sensor is immune to electromagnetic interference (EMI) that often compromises electronic sensors. It is relatively straightforward, inexpensive to produce, lightweight, small in size and self-referencing with a linear response. It is also ideally suited for measuring temperature and stress in potentially explosive environments because of its passive nature. FBGs can also be made sensitive to pressure, curvature, displacement, load and ambient refractive index.

**Figure 2.**
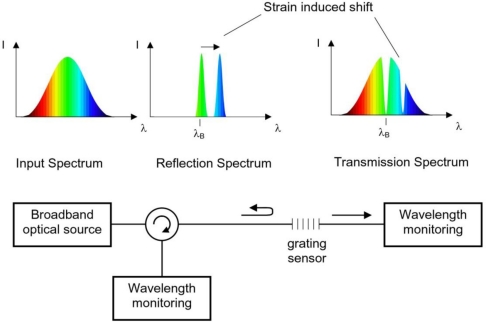
Basic Bragg grating-based sensor system with transmissive or reflective detection options.

The sensing function of an FBG originates from the sensitivity of both the refractive index of the optical fiber and the grating period within the fiber to externally applied mechanical or thermal perturbations. As the light reflected from the Bragg grating is dependent upon the spacing of the index modulation *Λ_G_* and the refractive index *n_eff_*, the strain field affects the response of the FBG directly, through the expansion and compression changes of *_G_* and through the strain-optic effect, *i.e.*, the strain-induced change in the glass refractive index. A schematic of a basic Bragg-grating based sensor system is shown in Figure 2.

The temperature sensitivity of the FBG is mainly due to the thermo-optic effect *i.e.*, temperature induced change in the glass refractive index and to a lesser extent, on the thermal expansion coefficient of the fiber. Thus, *λ_B_* shifts by an amount *Δλ_B_* in response to strain ε and temperature change *ΔT* by [3]:

(1) $$\frac{\Delta \lambda_{B}}{\lambda_{B}}=P_{e}\varepsilon +\left[P_{e}\left(\alpha_{s}-\alpha_{f}\right)+\zeta \right]\Delta T$$

where *P_e_* is the strain-optic coefficient, *α_s_* and *α_f_* are the thermal expansion coefficients of any fiber bonding material and of the fiber itself, respectively and ζ is the thermo-optic coefficient. Because FBGs can be written to have different resonant wavelengths, they can be multiplexed into a sensor web where different stresses or temperatures can be measured at different locations along the optical fiber length. Making use of this capability, Bragg grating sensors have been integrated into civil structures, aircraft, naval ships, oil pipelines *etc*. as ‘smart skin’ sensor webs to measure ‘*in situ*’ temperature and stress of these structures. At telecommunication wavelengths near room temperature, *λ_B_* varies with temperature at approximately 10 pm/°C.

## 2. Grating Types for Harsh Environmental Sensing

There are several different categories of fiber Bragg gratings which result from different processes. Recently a new classification system was proposed which is based on the formation mechanism [4]. Where appropriate, this nomenclature will be followed here.

### 2.1. Type I Gratings

Often referred to as standard gratings, Type I gratings were initially formed in germanosilicate fiber, at least for small index changes, by a single UV photon absorption process that excites oxygen deficiency defect centers (ODC) with absorption bands around 244 nm [5]. For large index changes, it is likely that defect formation is also accompanied by densification of the glass matrix [6].

Type I gratings exhibit a positive change in the refractive index and a temperature dependent decay which can be characterized using a power law [7]. This decay is the result of the thermal depopulation of trapped excited states that are created during the grating formation. At elevated temperatures, carriers in the shallowest traps can absorb enough energy to escape and return to the ground state. The remaining carriers are thus associated with more stable states which can also relax to the ground state if the temperature is elevated further. As the most unstable carriers decay at lower temperatures, type I gratings are typically annealed at temperatures higher than their designed operating temperature in order to obtain long term stability in their reflectivity. In terms of their applicability within a harsh environment (temperatures >450 °C) Type I gratings are unsuitable as most of the refractive index change is annealed out at these temperatures.

### 2.2. Type II Gratings

Using high peak power pulsed ultraviolet laser sources, such as Krypton Fluoride-based excimer lasers, high reflectivity gratings (<99%) have been inscribed with a single laser pulse [8]. These gratings result from a threshold dependent multiphoton ionization process similar to laser induced damage in bulk optics, hence the gratings are often referred to as ‘damage’ gratings. Such gratings are stable at temperatures over 1,000 °C and have been used to fabricate grating arrays while the fiber is being pulled on the draw tower [9]. The single shot exposure tends to produce grating structures which can suffer from significant scattering loss. The damage like process also has the tendency to reduce the reliability and mechanical strength of the fiber.

### 2.3. Type In (formerly Type IIA)

When UV-laser induced gratings are written in highly stressed Ge-doped core fibers, it is observed that under certain conditions, a Type I grating will grow and then decrease in strength as the grating saturates. Continued exposure results in secondary grating growth that exhibits a steady blue shift in the spectral response hence a negative refractive index change [10]. With optimized fabrication conditions grating Type In grating structures can be fabricated that are stable up to 700 °C [11]. The mechanism associated with the final index change is generally agreed to involve some sort of stress relief within the fiber.

### 2.4. Chemical Composition/Regenerated Gratings

The photosensitivity required for Type I grating formation can be enhanced through a process called hydrogen loading, where the optical fiber is exposed to high pressure hydrogen gas at room temperature [12]. Hydrogen gas permeates the glass matrix until the glass is saturated. Once the fiber is loaded, UV irradiation results in the hydrogen dissociation and the formation of Si-OH or Ge-OH defects resulting in increased levels of achievable index change. After UV inscription and out diffusion of unreacted hydrogen from the fiber, careful annealing of the grating at an elevated intermediate temperature of 600–700 °C results in interstitial diffusion of hydroxyl groups to form water molecules within the glass that become highly stable thermally [13]. The annealing at intermediate temperatures results in the erasure of the Type I seed grating however further heating at higher temperatures generates a new grating structure at a longer wavelength. The index modulation of this new grating is significantly less than the original seed grating by about an order of magnitude. It is stable at high temperatures however and can be repeatedly cycled to temperatures above 1,000 °C [14,15]. The remnant index modulation is typically less than 10^−4^ which results in a low reflectivity grating if its length is less than 1 cm. Because of its low index modulation, it is less susceptible to scattering losses that are often associated with Type II gratings. Recently it was shown that hydrogen need not be present during the laser inscription of the grating but during the thermal regeneration [16]. So it is still unclear as to what is the exact mechanism that is responsible for the process.

### 2.5. Femtosecond Pulse Duration Infrared Laser Induced Gratings

The ultrahigh peak power radiation generated by femtosecond pulse duration infrared (fs-IR) laser systems has been used to induce large index changes in bulk glasses for the fabrication of imbedded waveguides [17]. The mechanism for this laser induced index change is significantly different from the UV photosensitivity mechanism that is dependent upon color-center formation. The fs-IR laser approach is thought to result from a non-linear multiphoton absorption/ionization process leading to material compaction and/or defect formation depending on the intensity of the exposure. Above the material-dependent ionization threshold intensity *I_th_*, multiphoton ionization (MPI) induced dielectric breakdown likely results in localized melting, material compaction and void formation causing an index change that is permanent up to the glass transition temperature *t_g_*, of the material. The properties of the resultant index change are similar to those of Type II gratings induced by nanosecond pulse duration UV lasers but with one important difference. Because of the ultrashort duration of the femtosecond source, the induced dielectric breakdown is rapidly quenched upon cessation of the beam, hence there is virtually no propagation of ‘damage’ beyond the irradiated zone. This can produce grating structures with much better spectral performance than their UV counterparts.

Below *I_th_*, another regime of induced index change has been observed that can be erased by annealing with temperatures below *t_g_* [18]. In the regime below *I_th_*, multiphoton absorption likely results in defect formation and material compaction similar to that seen for Type I UV-induced index changes in photosensitive germanosilicate glasses.

Presently there are two main approaches to inscribing Bragg gratings with fs-IR laser sources. The first approach to be demonstrated, utilized a specialty phase mask that was precision etched to maximize coupling of the incident IR laser radiation into the ±1 orders [19]. Gratings were written in standard telecom fiber and then later in ‘non-UV photosensitive’ pure silica core fibers [20]. The phase mask-generated sinusoidal interference field results in a non-sinusoidal modulated FBG structure due to the nonlinear induced index change processes [21]. Using the phase mask approach, both Type I and Type II index changes can be induced in silica based glass fibers [21].

**Figure 3.**
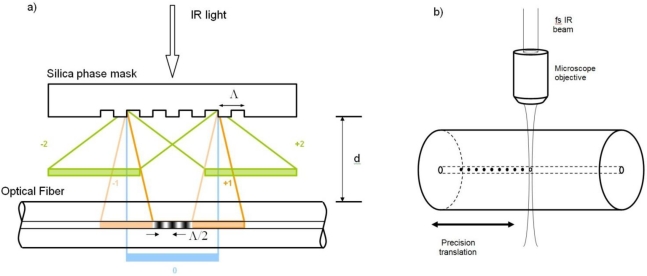
**(a)** Schematic of fs-IR phase mask inscription of a FBG; **(b)** Schematic of the point-by-point approach to FBG inscription.

The second approach to fs-IR laser induced FBGs utilized a ‘point by point’ writing technique where single pulses from the fs-IR laser were focused within the core region of the optical fiber using a powerful microscope objective. Highly localized changes to the refractive index were generated with each pulse to create each plane of the fiber grating. Subsequent planes were made in a step and repeat fashion by translating the beam using sophisticated high-resolution mechanical translation stages [22]. As the index changes resulted from intensities greater than *I_th_*, the grating structure consisted of material compaction surrounding void formation at each grating plane [23]. The resulting structure had similar thermal stability to Type II UV gratings [24]. A schematic of each of the two approaches is presented in Figure 3.

Unlike unsaturated UV-induced gratings, FBGs induced with a fs-IR laser either with a phase mask or with the point-by point method not only possess the fundamental Bragg resonance λ*_B_* but higher order resonances as well, such that:

(2) $$m\lambda_{B}=2n_{\mathrm{eff}}\;\Lambda_{G}$$

where *m* is the harmonic order number, *n_eff_* is the effective index of the guided mode and *Λ_G_* is the grating pitch.

The true advantage of the fs-IR laser approach to grating fabrication is that it is not limited to silica based UV-photosensitive fibers. The fs-IR approach to grating fabrication can be utilized in almost any waveguide that is transparent to the low signal infrared radiation of the writing source: silica and non-silica based glass waveguides, photonic crystal and crystalline waveguides and fibers [25]. From the perspective of grating structures for harsh environmental sensing, increasing the writing intensity beyond *I_th_* of the waveguide in question, results in a structure that is thermally stable approaching the glass transition temperature of the waveguide which for silica based fibers is >1,000 °C and for sapphire waveguides is >1,750 °C.

## 3. Harsh Environmental Sensing Applications of FBGs

### 3.1. High Temperature

**Figure 4.**
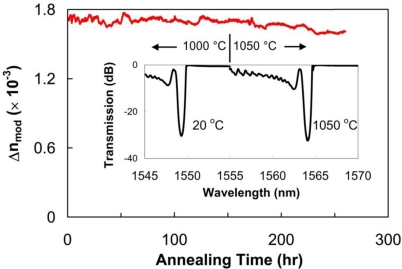
Grating reflectivity expressed as its index modulation (*Δn*) for a thermally stable grating (red) as a function of time. **(Inset)** Grating spectrum as a function of temperature.

High-temperature resistant FBGs, such as regenerated gratings or those written with femtosecond 800 nm laser pulses—will open up opportunities in sectors where harsh environments exist such as power plants, turbines, combustion, and aerospace. Attributes of FBGs written with fs-IR radiation above *I_th_* in both Ge-doped SMF-28 from Corning and pure silica core (fluorine doped silica clad) fibers were studied over a longer term [26]. Bragg gratings with large index modulations (*Δn*) were inscribed in both fiber types which were then heated to 1,000 °C in 100 °C increments, 1 hour intervals within a tube furnace. The FBGs were loosely placed to avoid the application of external stresses. Once 1,000 °C was reached, the grating temperature was maintained at 1,000 °C for 150 hours while monitoring the FBG transmission spectra (see Figure 4). There was no noticeable degradation of the grating strength for the duration of the test and the grating maintained Δ*n* = 1.7 × 10^−3^. Spectra taken initially at room temperature and after 100 hours at 1,050 °C are shown in inset of Figure 4. The increase in Δ*n*, noted by the slight elevation in grating reflectivity, is likely a result of the two kinds of index change being written simultaneously. The peaks of the complex interference pattern are sufficiently intense to ionize the glass in the fiber producing an index change that is durable with temperature. In the valleys of the interference pattern, the intensity is below the Type II threshold, however some Type I index change is generated. As the device is annealed, the permanent Type II index change remains while the annealable Type I index change is erased resulting in a higher Δ*n* contrast. The temperature of the FBG was then increased and kept at 1,050 °C for 100 hours during which the Δ*n* decreased slightly from 1.7 × 10^−3^ to 1.6 × 10^−3^. A drift of the Bragg resonance to longer wavelengths of 0.2 nm was detected at the end of experiment. When the fiber is pre-annealed at high temperatures in order to relax residual stresses, Type II fs-IR FBGs are operable up to 1,200 °C [27]. This reflectivity is an order of magnitude higher than what is obtainable using type II UV or regenerated gratings.

For operation in a high temperature environment, an important issue for any silica-based fiber optic sensor is that of sensor packaging. At temperatures close to or above 1,000 °C in air, unpackaged standard silica single mode fibers loose almost all of their mechanical strength. While the fibers themselves survive hundreds of hours at 1,000 °C [26] when left untouched, any subsequent handling of the fiber after the test is not possible as the fiber becomes extremely brittle. Obviously optical fibers experience severe mechanical degradation when tested in oxidizing atmospheres at high temperature.

Protecting the fiber from exposure to oxygen at high temperature could be achieved by using a suitable package that itself survives the high temperature. The most obvious choice would be a coating on the fiber that is applied after the gratings are written or by writing through the existing protective coating. Metallic coatings are preferred for higher temperature applications, with gold coatings rated for the highest temperature operation of 700 °C, however this rating is not suitable for temperatures close to 1,000 °C [28].

**Figure 5.**
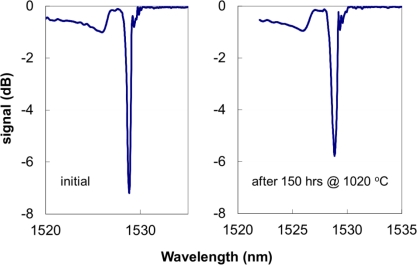
Initial and final room temperature transmission spectra of the FBG in the fiber cane. Final spectrum taken after one week at 1,020 °C.

Using the fs-IR laser approach, a Type II grating was made in a fiber cane, *i.e.*, a single mode optical fiber with a 400 μm cladding [29]. Such a device is considered as a self-packaged FBG device for applications at or above 1,000 °C [30]. The 400 μm clad fiber maintained enough mechanical integrity to be easily handled even after 150 hours above 1,000 °C. Room temperature spectra taken before and after the 150 hours of annealing are shown in Figure 5. In order to test the mechanical strength after the long term annealing, the fiber cane was placed in a tube furnace in air with only the central section of the furnace set at 1,020 °C. The two side sections of the furnace were set at 400 °C in order to preserve the mechanical integrity of the fiber outside the central high temperature zone so that a pull test on the section of the fiber subjected to the 1,000 °C temperature could be performed. The fiber was kept at 1,020 °C for about 100 h, then the temperature was slowly ramped down to room temperature. After the fiber was removed from the furnace it was proof tested to determine its mechanical state after the high temperature annealing. The fiber was suspended in a vertical position from a support and weights were added to the fiber in 50 gram increments with a 60 second delay time for the application of each new weight. The fiber was tested up to 500 grams force which for the 400 μm diameter corresponds to ∼20 kpsi maximum stress. The pristine fiber was proof tested by the manufactures to 100 kpsi. Although the fiber is stiffer than 125 μm diameter SMF fiber, it can normally accept bending radii of less than 10 cm making it suitable for the majority of sensing applications.

**Figure 6.**
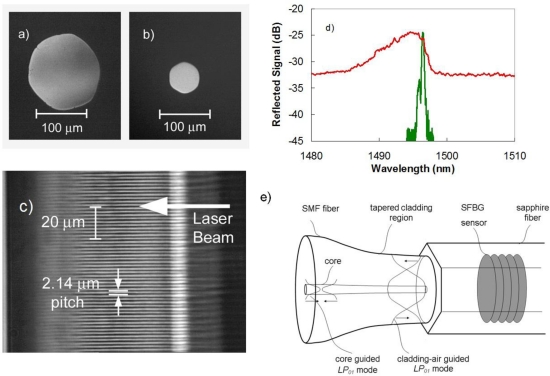
Cross-sections of commercially available **(a)** 125 *μ*m and **(b)** 60 *μ*m diameter sapphire fiber are shown; the grating structure inscribed in 150 *μ*m diameter fibre is shown in **(c)**; the corresponding multimode reflection response is shown by the red trace in **(d)**; when using the single-mode field expander shown in **(e)**, the single-mode reflection spectrum shown in green in **(d)** is obtained.

For temperatures >1,200 °C, silica based optical fibers are no longer appropriate. The most successful optical fiber used for high temperature sensing applications is the single crystal sapphire fiber that has a glass transition temperature *t_g_* of ∼2,030 °C. Unlike conventional single mode optical fibers, sapphire fibers are made in the form of rods absent a cladding layer which makes the sapphire waveguides highly multimode and sensitive to bending losses and mode conversion. With fiber diameters commercially available, beam propagation within the fiber is highly multimode at the 1,550 nm telecommunication wavelengths. An example of available fiber diameters is shown in Figure 6(a). Present sapphire fiber sensors are mostly based on Fabry-Perot structures within the fiber producing a broadband interferometric signal that varies with temperature [31]. Such devices are used effectively as point sensors.

Femtosecond laser inscribed sapphire FBGs (SFBGs) are naturally multimode devices producing a reflection response that is not as sensitive to temperature and strain as the single mode FBG sensor counterparts [32]. The red trace in Figure 6(d)) presents an example of a typical multimode reflection response from a SFBG sensor when interrogated with a multimode coupler and white light source. The multimode reflection spectrum observed with the retro-reflective SFBGs is characterized by a large bandwidth having a complicated structure that consists of a superposition of different modes reflected by the grating. It is preferable to produce a narrowband single mode response, as it increases the sensitivity of the spectral response to changes in temperature and strain. In order to arrive at a single mode response from the multimode SFBGs, they were probed by using an adiabatic fiber taper to expand the ∼10 μm diameter single mode into a fundamental mode approaching the diameter of the sapphire fiber (as shown in Figure 6(e)) [33]. The fundamental guided mode of the sapphire waveguide is excited producing a single mode reflection response (green trace Figure 6(d)). In this fashion, single mode reflection responses consistent with existing FBG sensor array interrogators can be generated. SFBGs exhibit no degradation of the grating strength at high temperatures up to 1,745 °C [34]. The SFBG has definite advantages over other sapphire fiber sensors that rely on Fabry-Perot etalons at the fiber tip. Unlike Fabry-Perot sapphire sensors, SFBG sensors with their discrete resonant wavelength could potentially be used as distributed optical sensor arrays up to 2,000 °C.

Gas turbine monitoring is an example where high temperature optical sensing would be useful. Accurate measurement of hot gas working temperatures within a turbine is the critical control parameter that is essential for safe, reliable, efficient, and cost-effective operation of the gas turbine. Inhomogeneous combustion can lead to overheating and considerable damage to turbine blades, however accurate measurements of the blades and vanes within a gas turbine are very difficult [35]. By probing gas temperatures in the turbine exhaust path, temperature distributions within the combustion chamber can be evaluated. Recently several types of high temperature FBG sensors were evaluated for monitoring of hot gas turbine components by placing them on a heat shield tile mounted on the side of the combustion chamber exhaust [36]. Several of the gratings were erased at various temperatures depending on the fabrication technique used: UV-laser induced draw tower gratings annealed out at 250 °C, weak and strong excimer laser induced Type I gratings at 450 °C and 700 °C respectively, UV Type II gratings at 900 °C. Fs-IR laser induced Type II gratings and regenerated gratings were observed to have short term stability at 1,000 °C, however softening of the fused silica was observed to commence at 850 °C which resulted in a drift in the grating response. At 800 °C, only the femtosecond infrared gratings were reported to have no measurable drift even after a duration of several months.

### 3.2. High Radiation

For nuclear applications, electronic transducers and gauges are often unsuitable in nuclear environments especially when intense nuclear radiation is accompanied by elevated temperatures, chemical contamination and high levels of electromagnetic interference. Neutron bombardment results in damaging effects to materials, for example thermocouple output will drift under intense radiation as the two metals forming the thermocouple gradually transmute into different elements.

UV-laser inscribed fiber Bragg grating sensor arrays based on Ge-doped optical fiber have been demonstrated to be radiation tolerant in low flux nuclear environments, even for extended periods of time [37]. However in high flux environments, Ge-doped optical fibers are susceptible to radiation induced attenuation (RIA) which after a period of time will render the fibers opaque. The RIA process is similar to the defect formation mechanism that is occurs during UV Type I grating inscription in Ge-doped fibers. For reliable strain and temperature measurements it is therefore necessary to inscribe gratings in radiation hardened fibers that are resistant to RIA, such as fibers with pure or fluorine-doped silica cores [38]. Because these fibers, by their design, are inherently non-photosensitive, standard grating writing techniques are ineffective. Gratings were easily inscribed in highly radiation harden F-doped fiber [39] using the fs-IR approach with a phase mask, both in the Type I and Type II regime [40]. Very little change was observed in the spectral quality of the grating even after a dosage of γ-radiation of 100 kGy.

### 3.3. Multi-Parameter Sensing in Harsh Environments

For FBG sensors, the resonant Bragg wavelength of the grating, *λ_B_*, is the primary sensing parameter that is monitored. Unfortunately it is often difficult to discriminate between different effects, for example temperature and stress, with a single FBG since different effects can impact simultaneously on *λ_B_*. Often another Bragg grating in a favorable arrangement is used for each of the parameters involved in a particular case, but this procedure will result in a very complicated sensing configuration. In order to avoid the multi-fiber solution to multi-parameter sensing problems, a variety of other alternatives have been used. Techniques to discriminate sensing parameters include double superposed grating structures with different *λ_B_* [41], FBGs in fiber with dissimilar diameters [42], utilization of the second order diffraction from a saturated first order 1,550 nm grating [43], FBGs in birefringent fibers [44], *etc*. These techniques were developed mainly for the particular case of strain and temperature discrimination. A comprehensive summary of these methods is presented in the review paper [45].

**Figure 7.**
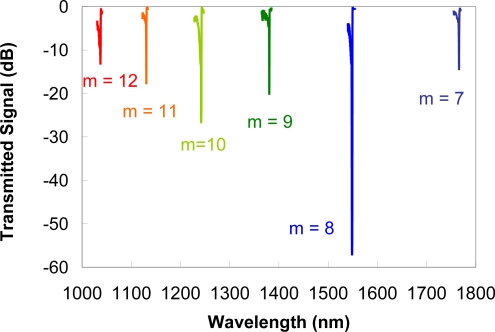
High-order spectra of a 4.28 μm pitched Type II Bragg grating made in a 980 nm cut-off fiber.

For high temperature environments below 1,000 °C, Type II gratings in silica fibers can be effective multiparameter sensors. Whether written with pulsed UV lasers or femtosecond IR lasers, the non-sinusoidal profile of the index modulation results in higher order resonances [20,46] as previously noted in Equation (2). For a standard grating in low wavelength cut-off telecommunication fiber, the grating pitch needed for a fundamental Bragg resonance at 1,550 nm is 535.5 nm. The fabrication of a fs-IR laser written Type II grating with a pitch *Λ_G_* = 4.184 μm, 8 times longer than the needed fundamental pitch, produced six high order resonances that are observable when viewing the transmission of the grating in the wavelength range from 1 to 1.8 μm [47]. The multiple order resonances from the grating are depicted in Figure 7. From Equation (2), the order *m* of these resonances range from 7 to 12. The strain and temperature characterization of each resonance in this wavelength range was made and the temperature and the strain coefficients were evaluated. Wavelength shifts of the resonances due to temperature or strain were different for each grating order. In this way using a single grating, the cross sensitivity of temperature and strain can be removed by monitoring the high order resonances.

An interesting approach to multiparameter sensing has recently been developed, which exploits the cladding mode resonances that are generated when a Bragg grating is tilted slightly (10°) with respect to the fiber axis [48]. The cladding mode resonances are not core-cladding guided but cladding-air guided and can therefore interact with the environment surrounding the fiber. All the resonances, both core and cladding, have the same temperature dependence near room temperature in terms of wavelength shift however the strain dependence of high order cladding modes differs from that of the core mode. Hence a dual temperature/axial strain sensor can be obtained from a single tilted grating. Since these gratings are based essentially on Type I UV formation properties, they would not be effective in high temperature environments. Because regenerated gratings typically have low index modulation, the weak resonances would not be ideal for sensor operation. To date, a regenerated tilted Bragg grating sensor has not been fabricated.

By inscribing a grating with a fs-IR laser using the point by point technique in such a way that the index change is localized near the core-cladding interface of the fiber, it is possible to couple much of the guided mode into cladding modes that behave in a similar way to the cladding modes generated by a tilted grating [49]. The point-by-point grating structure consists of micro-void spots that make up each grating plane. As such, these structures are thermally stable up to at least 1,000 °C. Such a device could therefore conceivably be used as a multiparameter sensor in high temperature environments. To date however, such a structure has only been demonstrated as a multiparameter sensor at more modest temperatures (<75 °C) [50].

**Figure 8.**
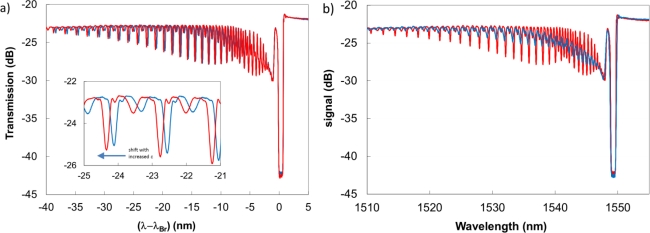
**(a)** Offset transmission spectra of Type II fs-IR FBG at 19 °C, subjected to tensile loads of 19 g (blue) and 509 g (red). Measured using a tunable laser with 1 pm resolution; **(b)** Change in cladding mode structure as a function of temperature, 19 °C (red), 800 °C (blue).

Type II gratings written with the femtosecond IR laser and the phase mask possess strong cladding mode structures [20,21], but not as strong as those associated with tilted gratings [48] or fs-IR point-by-point gratings written asymmetrically within the core [49]. Characterization of the core and cladding mode responses associated with untilted Type II fs-IR gratings as a function of temperature and strain were measured up to 800 °C [51]. As with both the tilted UV and the fs-IR asymmetric point-by-point gratings, a difference between strain dependent wavelength change of the core and cladding modes was observed (see Figure 8(a)). However at higher temperatures (>800 °C) coupling into the cladding modes was reduced making it difficult to measure discrete modes. (see Figure 8(b)).

Above 1,200 °C multiparameter optic sensors cannot be made with silica fibers. There are several instances where measuring both temperature and strain in a high-temperature environment is important, for example, inside jet engines and turbines for electric power generation using natural gas. For these temperatures, sapphire based FBG sensors have been demonstrated for temperature measurements [32–34]. At very high temperatures (>1,000 °C) thermal blackbody radiation produces a strong signal background that will reduce the signal to noise ratio of a multimode Bragg grating reflection [34]. By exciting the fundamental mode of the sapphire waveguide, the single mode grating reflection improves the signal to noise ratio. Monitoring of blackbody radiation with sapphire fibers is well known [52]. By taking advantage of the improved signal to noise ratio afforded by fundamental mode excitation of the sapphire FBG and the known blackbody radiation spectrum as a function of temperature, a dual parameter strain/temperature sensor in sapphire fiber was realized [53]. The signal level of thermal blackbody radiation was monitored as a temperature reference, in order to allow for the portion of the wavelength shift dependent on temperature to be decoupled from the strain.

### 3.4. High Pressure Sensing

When exposed to high pressures, Type I FBGs undergo a negative wavelength shift that is proportional to the level of hydrostatic pressure [54]. The wavelength shift is modest and is on the order of −3 pm/MPa but was demonstrated to change in a linear fashion to pressures up to 10,000 psi (70 MPa). By using elastomer and polyurethane fiber coatings, the sensitivity of the gratings to pressure could be increased 20 fold [55]. More recently a carbon fiber laminated composite structure was adhered to an FBG which further enhanced its sensitivity by 3 orders of magnitude and was demonstrated at pressures up to 70 MPa [56]. In these cases, pressure measurements are conducted at or near room temperature.

If there is a requirement to measure high pressures as well as high temperatures simultaneously, then a multiparameter pressure/temperature sensor based on Type I grating structures is undesirable at temperatures above a few hundred °C. Using a side hole fiber geometry, simultaneous measurements of high pressure and temperature were performed [57]. The birefringent nature of the fiber resulted in two polarization dependent resonances of the Type I Bragg grating. With increasing pressure within the side holes, a reduction in the separation of the polarization dependent resonances was observed. Any changes in temperature were observed by identical wavelength shifts of both resonances.

Using the same kind of side hole fiber geometry, thermally stable fs-IR laser induced Type II and regenerated gratings were inscribed in microstructured side-hole fiber in order to produce a sensor that could simultaneously monitor temperature and pressure in harsh environments [58,59]. These sensors were demonstrated to operate well in pressure ranges from 15 to 2,400 psi (0.1 to 16.5 MPa) and at temperatures up to 800 °C. As well the core region of the fiber was located such that the guided mode would have some evanescent coupling into the side-hole region. With a grating in place, this structure could be used to measure the refractive index of materials or fluids within the side-hole.

### 3.5. High Pressure Hydrogen Detection

There has been considerable interest recently in the use of hydrogen especially in the domain of energy production and as an alternative fuel for combustion engine vehicles in the automotive sector. Hydrogen itself should not generate pollutants in the combustion process and can be produced from renewable sources. While hydrogen fuel cells are most likely the most promising large industrial application, hydrogen is also being investigated for petrochemical processes and other uses. Hydrogen is usually stored under high pressure. In the nuclear industry, next generation reactors such as the supercritical-water cooled reactors (SCWR) and the very-high-temperature reactors (VHTR) are seen as potentially being efficient methods for mass production of hydrogen, however high pressures and temperatures are required in these processes (25 MPa, 1,000 °C). As a result, hydrogen sensors will be required for hydrogen detection in different ambient conditions and harsh environments. They will need to be small, sturdy, inexpensive, reliable over a large temperature range, reproducible with long-term stability and operable in potentially explosive environments.

Specialized FBG sensors based on the application of a layer of palladium on the surface of the fiber were investigated for hydrogen detection in the range of 1–4% volume concentration. These sensors can be monitored by the spectral shift of the Bragg wavelength that is caused by strain resulting from volume changes of the palladium layer [60] or by changes in the palladium refractive index [61] in the case of side polished fibers that evanescently couple into the palladium layer. To increase the sensor response time, the fiber may be heated. While being very sensitive to small concentrations of hydrogen, the palladium–hydrogen system is dependent on the hydrogen pressure and temperature, which can be in two crystallographic phases called α and β, separated by a phase transition. It is necessary to operate the sensor in the temperature-pressure domain of either of these two phases [62].

The hydrogen loading process, which is used to increase the photosensitivity of Ge-doped silica waveguides to UV light [12], can be exploited as a hydrogen sensor. The loading process typically requires placement of optical fibers for 1–2 weeks in an atmosphere of H_2_ at room temperature and ∼2,000 psi or for 1–2 days at higher temperatures of 80 °C–100 °C. The hydrogen does not react chemically with the fiber, but is in ‘solution’ with the glass matrix. Upon removal of the optical fiber from the high pressure H2 environment, the hydrogen within the fiber immediately starts to out-gas [63]. The presence of the H_2_ within the fiber results in an increase in the refractive index of the fiber core, which is dependent upon the hydrogen partial pressure and temperature. Such loading typically results in a 1 nm wavelength of the Bragg resonance to longer wavelengths while the fiber is pressurized. Out gassing will result in the Bragg grating resonance eventually shifting back to its original wavelength. While the time constant of this process is too long for a sensing process, it can be greatly reduced by using specialty optical fibers such as side hole or photonic crystal fibers that can bring the hydrogen in closer proximity of the fiber core which can be in more direct contact with the ambient hydrogen gas. Out diffusion rates of high-pressure hydrogen gas from photonic crystal fiber cores using Bragg gratings were recently reported [64].

**Figure 9.**
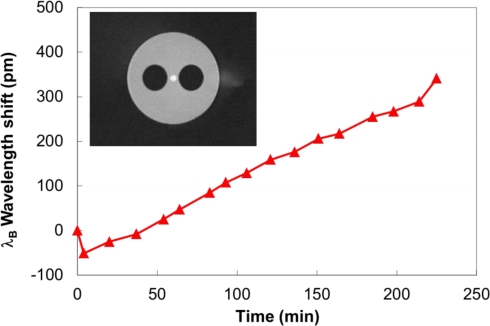
Wavelength shift with FBG exposure to 1,000 psi of H_2_ at room temperature. **(inset)** Photo micrograph of the side-hole fiber used.

An FBG was fabricated with fs-IR radiation in a single mode side-hole fiber similar to what is shown in the inset of Figure 9 [65]. The room temperature shift in the Bragg resonance when exposed to 1,000 psi of H_2_ is shown in Figure 9. Immediately after applying high pressure to the chamber the resonant Bragg wavelength shifted toward shorter wavelength by ∼50 pm. Since the diffusion process is not that rapid, this negative wavelength shift is due to the applied high pressure [54]. In the first 15 minutes, a positive 25 pm wavelength shift was measured due to the hydrogen diffusion into the fiber core. The wavelength shift continued for the duration of the experiment (>6 hours) until the hydrogen was evacuated. Immediately after evacuation of the hydrogen, a large positive wavelength shift (50 pm) was detected corresponding to the reduction of the applied high pressure. The out diffusion process that follows the removal of the fiber from the chamber results in a negative wavelength shift of the grating resonance with a rate of change of the wavelength that is about the same but in the opposite direction.

As in the case of palladium based sensors, the rate of hydrogen diffusion into the fiber core can be increased by ambient pressure and fiber heating. Since only the hydrogen will diffuse into the silica, the method described above can be used to measure partial pressure of hydrogen in gas mixtures at high pressure. Although not presented here, the femtosecond laser approach to grating inscription is ideally suited for pure silica core photonic crystal fibers [66]. With guided light evanescently coupled into the cladding holes, detection of diffused hydrogen from the cladding holes is more rapid [64].

### 3.6. High Reliability FBGs for High Strain Measurements

For structural integrity monitoring using FBG sensor arrays, the capability to measure high strain levels is very desirable. The standard UV approach to grating inscription requires that the protective polymer jacket, which is highly absorbing in the UV, be removed prior to and replaced after the FBG fabrication. Coating removal and replacement are time-consuming processes that threaten the mechanical integrity of the fiber reducing the maximum value of measurable strain.

Using the fs-IR laser and the phase mask technique, FBGs were successfully inscribed through the acrylate coating of standard SMF-28 fiber [67] and high numerical aperture (high Ge-doped) fiber [68], after the fiber’s photosensitivity to fs-IR radiation was enhanced by hydrogen loading [69]. Index modulations of up to 1.4 × 10^−3^ were induced in the high NA fibers with strengths remaining at 75% to 85% of the pristine fiber value. For strain sensing applications however it is desirable to have the FBG in polyimide-coated bend insensitive fibers, as the thin polyimide coating better transfers mechanical information to the fiber than the thicker and softer acrylate coatings. Using the same approach but in polyimide-coated bend-insensitive high NA fibers, index modulations of 1 × 10^−4^ were generated with strengths remaining at ∼50% of the pristine fiber value [70].

### 3.7. Oil and Gas Applications

For applications in the Oil and Gas sector, fiber optic sensors are hampered by fiber losses resulting from ingress of hydrogen gas that occurs at the elevated temperatures within the downhole environment. This is especially true for standard Ge-doped telecom fibers. Employing pure silica core fibers that do not suffer from hydrogen-induced attenuation can mitigate these effects [71], however FBG based distributed sensing arrays are extremely difficult to produce in pure silica core fiber using conventional UV-laser based inscription. The approach of using high photonic energy 193 nm radiation in pure silica core fibers [72] requires very lengthy exposure times which degrade fiber reliability and are not practical from a production standpoint. Using the fs-IR approach, high *Δn* FBGs can be easily written in pure silica optical fibers that are resistant to hydrogen induced loss [20]. The use of such fibers is key for improving sensor array performance to monitor for example steam-assisted gravity drainage in the oil and gas industry.

## 4. Conclusions

In this paper, some of the recent developments in fabrication and application of fiber Bragg gratings for extreme environment sensing have been presented. For temperatures less than 1,000 °C, FBGs made in silica based fibers either through femtosecond infrared laser exposure or by the grating regeneration process can result in spectral responses which are stable at high temperatures. When exotic grating or fiber geometries are employed, multi-parameter sensing from a single grating element can be realized. Gratings written in glasses such as pure silica, or radiation hardened fluoride-doped silica can be used for sensors in the oil and gas, or nuclear industries where losses in standard optical fiber due to hydrogen ingress or ionizing radiation can significantly reduce sensor lifetime. Above 1,200 °C extreme temperature with Bragg gratings is relegated to sapphire optical fiber. In the case of sapphire FBGs, these robust devices are suitable for harsh combustion environments such as jet engines, coal gasification reactors, and natural gas turbines for electrical power generation.
